# Severe acute respiratory syndrome-coronavirus 2 in domesticated animals and its potential of transmission: A meta-analysis

**DOI:** 10.14202/vetworld.2021.2782-2792

**Published:** 2021-10-27

**Authors:** Yos Adi Prakoso, Chylen Setiyo Rini, Yuli Purwandari Kristianingrum, Nurul Hidayah, Dyah Widhowati, Miarsono Sigit

**Affiliations:** 1 Department of Pharmacology, Faculty of Veterinary Medicine, University of Wijaya Kusuma Surabaya, East Java, Indonesia; 2 Integrated Laboratory, Faculty of Health, University of Muhammadiyah Sidoarjo, East Java, Indonesia; 3 Department of Pathology, Faculty of Veterinary Medicine, University of Gadjah Mada, Yogyakarta, Indonesia; 4 Department of Microbiology, Faculty of Veterinary Medicine, University of Wijaya Kusuma Surabaya, East Java, Indonesia; 5 Department of Veterinary Reproduction, Faculty of Veterinary Medicine, University of Wijaya Kusuma Surabaya, East Java, Indonesia.

**Keywords:** asymptomatic, domesticated animals, pet, severe acute respiratory syndrome-coronavirus-2, transmission

## Abstract

**Background and Aim:**

The coronavirus diseases-2019 (COVID-19) pandemic has caused a global lockdown, which has limited the mobility of the public, and thus, more time is spent with their pets. Unfortunately, many social media have blamed pet animals as a reservoir of severe acute respiratory syndrome-coronavirus 2 (SARS-CoV-2), the etiologic agent of COVID-19, triggering a panic abandonment of pets. However, no article has summarized the information regarding the role of pets as SARS-CoV-2 reservoirs. This study aimed to evaluate the role of pets as a reservoir of SARS-CoV-2 on the basis of research papers (i.e., animal model, surveillance, and case report) published in 2020.

**Materials and Methods:**

The review was conducted using articles from the PubMed database in 2020, using the keywords “COVID-19 in domesticated animals,” which were screened and analyzed. Only the data from research articles were mimicked and transformed to conduct a meta-analysis. The meta-analysis was conducted regarding the effects of inhabitation and viral shedding in pets. In this study, we used 95% confidence intervals.

**Results:**

A total of 132 papers in PubMed were related to the keywords, whereas only 12 papers were appropriate to answer the dynamics of the role of pets as the reservoir for SARS-CoV-2. Seven studies indicated the potential of cat-cat (4/7), human-cat (2/7), and human-dog (1/7) SARS-CoV-2 transmission. No study proved the presence of cat-human transmission. Another study showed that comingling did not affect SARS-CoV-2 viral shedding among a cat and dog. Furthermore, the viral shedding of cats and dogs caused asymptomatic manifestations and generated neutralizing antibodies within a short period of time.

**Conclusion:**

SARS-CoV-2 transmission is present in domesticated animals, especially in pet cats and dogs, and transmission occurs between animals of the same species (cat-cat). The reverse zoonosis (zooanthroponosis) was found from human to cat/dog (comingled) with asymptomatic clinical signs due to the representation of neutralizing antibodies.

## Introduction

The coronavirus is an enveloped RNA virus from the *Coronaviridae* family that can affect humans and animals. This virus causes enteric, respiratory, and systemic diseases in mammals [1]. The clinical appearance of the coronavirus varies depending on the host immunity and sensitivity in response to infection. The coronavirus has a spike protein on its virion surface. These spikes increase the attachment and fusion capabilities of the virus to host cells [2]. Several types of coronaviruses that have caused human infection are severe acute respiratory syndrome-associated coronavirus (SARS) in 2003 and Middle East respiratory syndrome in 2012. Both of these viruses are contagious and cause respiratory illness. A new coronavirus was discovered in 2019, known as a SARS-coronavirus 2 (SARS-CoV-2) [3].

SARS-CoV-2 has caused a global pandemic within a couple of months after it was first discovered in Wuhan, China, in December 2019. This virus has high morbidity and mortality among affected patients. John Hopkins University reported that the SARS-CoV-2 infection had reached more than 120 million people worldwide. This forced the state government to implement lockdowns in 2020 to prevent the massive transmission of SARS-CoV-2 [4]. These lockdowns limit the activity of people, including those infected (i.e., self-quarantine), to go outside except shop for daily needs. Both lockdown and self-quarantine increase the activity of society at home, including an increased rate of direct contact with pets [5].

SARS-CoV-2 infection has been reported in animals with symptomatic [6] and asymptomatic clinical signs [7]. Scientists and public health experts have suspected that pets can potentially transmit or act as a reservoir for SARS-CoV-2, because the other types of coronaviruses can transmit and infect animals as well. Several suspected animals are cats, dogs, rats, hamsters, and other companion animals. Moreover, a lot of social media posts have blamed pet animals as a reservoir for SARS-Cov-2, thus triggering the panic abandonment of some pets [8]. These uncontrolled issues without inputs from veterinary experts, virologists, or scientists can lead to discrimination of these companion animals [9].

To uphold animal welfare and prevent social panic, this study aimed to evaluate the role of pets as a reservoir of SARS-CoV-2 on the basis of existing research. The selected research paper includes animal model, surveillance, and case report published in 2020.

## Materials and Methods

### Ethical approval

The study is based on the published articles only and not related to live animals so, it does not require ethical approval.

### Study protocol

This study used the protocol described by the PRISMA statement to determine the preferred reporting items in a review and meta-analysis [10].

### Articles collection

We collected articles published in PubMed^®^ (https://pubmed.ncbi.nlm.nih.gov/), selected on the basis of the year of publication and suitability with the keywords. This study focused on articles published in 2020 only; other years of publication were excluded. Furthermore, we searched related articles using the keywords “COVID-19 in domesticated animals,” and the collected papers were classified as either a review or research (i.e., animal model, surveillance, case report, and *in silico* study). Each paper was screened for suitability on the basis of the title and abstract. All the titles and/or abstract should contain elements such as coronavirus diseases-2019, domestic animals, companion animals, animals, and pets (i.e., cat, dog, and ferret). Review papers and other papers without any relation to the keywords and elements were excluded and used only as supplementary material, if applicable.

### Eligibility criteria

The preferred reporting items had their content analyzed using several categories as follows: Type of study, host, duration of infection, diagnostic methods, viral shedding, histopathology, clinical signs, and potential of transmission. Studies were classified as either experimental study using animal models, surveillance, case report, or *in silico* study (computational study). The eligibility criteria were as follows:

1. The host indicator was determined on the basis of the types and numbers of animals used in the observation
2. The duration of infection was used to determine the period of infection from the first to the final days of the experiment
3. Diagnostic tools were used
4. Virus shedding, histopathology, and clinical signs were reported and correspond to the finding of the preferred reporting items
5. The potential of transmission was determined according to the claim of the previous study (whether it was potent or not), site, the shortest period where the virus was first detected, and the presentation of neutralizing antibodies against SARS-CoV-2
6. Correlation between several eminent factors from the previous studies, especially inhabitation, SARS-CoV-2 viral shedding, clinical signs, and the presentation of neutralizing antibodies.

### Data transformation

The data were transformed to mimic all the data from the included studies. The transformation was conducted using the data of companion animals and the test results of their specimens. Data collection focused on experimental studies that used natural infection, surveillance, and case report that were applicable for further analysis. The data collected from companion animals, with a focus on cats and dogs, included inhabitation (comingled or separated with the owners), viral shedding (positive or negative), clinical signs (with or without), and presentation of neutralizing antibodies against SARS-CoV-2 (positive or negative). The data were tabulated and scored using a simple scoring system (1: positive/with and 2: negative/without).

### Statistical analysis

Before the analysis, all the data were tabulated. Then, the data were analyzed using the Chi-square test. The analyses were conducted on the correlations between inhabitation and viral shedding, viral shedding, and clinical signs, and between viral shedding and the presentation of neutralizing antibodies against SARS-CoV-2. The risk ratio (RR) using a 95% of confidence interval (CI) was calculated. All the statistical analyses were conducted using statistical package for the social sciences version 16.0 (IBM SPSS, NY, USA) with a p=0.05. The rest of the data were analyzed using simple qualitative descriptive analysis.

## Results

### Correlated articles

**Figure-1:**
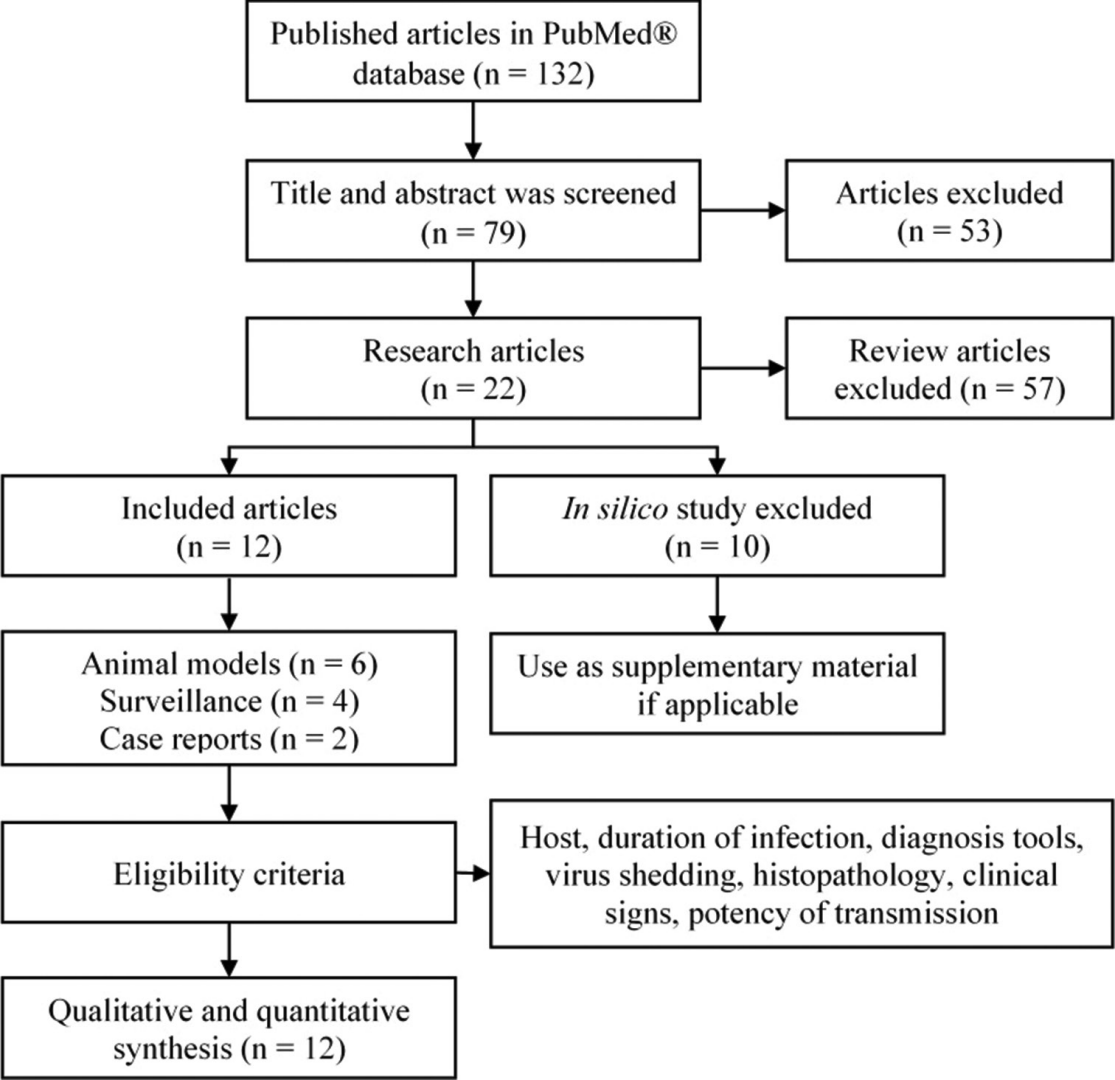
Flowchart of literature search.

A total of 132 articles were collected from PubMed® using selected keywords. The article was screened using the title and abstract. Then, 53 articles were excluded because of a mismatch of the title and abstract with the keywords. Furthermore, an additional 57 papers were excluded because of the types of articles; these consisted of 7.01% (4/57) letters to the editor, 5.26% (3/57) commentaries, and 87.71% reviews (50/57). Finally, 22 research papers matched the criteria. These papers were placed into advanced classifications for better analysis, including animal modeling either artificially or through natural infection (27.27%), surveillance (field study; 18.18%), case report (9.09%), and *in silico* study (45.45%) (Table-1). Furthermore, *in silico* studies were used as supplementary materials because this type of study only uses computational data such as the DNA sequences of SARS-CoV-2 among animals. The flowchart of the literature search is embedded in Figure-1.

**Table-1:** Number of paper merit to the criteria.

Classification	Total	Annotation
Article merit to keywords	132	-
Article merit to title and abstract	79	53/132 papers were excluded - Do not contain elements
Review paper	57	Excluded - Due to the article type (letter to editor, commentary, and review)
Research		
Animal model	6	Included
Surveillance	4	Included
Case report	2	Included
*In silico*	10	Supplementary material
Total paper merit to the criteria	12	Included

### Study characteristics

The characteristics of the included studies are presented below. These characteristics included type of study, species of animal, type of infection, period of infection, and summary of the results. In addition, there were more than 30 species of animals observed as the target of exploration for SARS-CoV-2. These included companion animals (cat, dog, and ferret), livestock (pig, sheep, horse, and cow), laboratory animals (rat, guinea pig, rabbit, rhesus, and monkey), poultry (chicken, duck, and goose), and wild animals. These animals were seen in the experimental studies (artificial and natural infection), surveys, and case reports (Table-2) [11-22].

**Table-2:** Detail of included studies.

Type of study	Animal species (number of animals used)	Types of infection	Period of infection (days)	Result	Reference
Animal model	Cat (10)	AI, NI	1-2	a. Asymptomatic clinical sign b. RNA virus was detected in respiratory swab until day 21, however, in non-respiratory organ start from day 3 to 21 c. Histopathology indicated mild-moderate lymphocytic neutrophilic adenitis until day 7, and no histopathological changes on day 21 d. SARS-CoV-2 RNA antigen was found in bronchi on days 4, 7, and absence on day 21 e. Virus neutralizing antibody found in days 5-21	[11]
Animal model	Ferret (5), cat (7), dog (7), pig (8), chicken (8), duck (8)	AI, NI	Varies from 1 to 10	a. RNA virus was highest detected from nasal and turbinate in ferret and cat b. NA virus was not detected from an oropharyngeal and rectal swab in pig, chicken, and duck, however, was detected in the dog (1/5)	[12]
Animal model	Cat (7), dog (3)	AI, NI	1 – 42	a. Dog and cat groups did not show any clinical signs b. Viral shedding was found in the cat’s nasal on day 3, and it was observed following oral exposure from 24 h PI c. Viral shedding was not found in the dog d. All cats showed moderate histopathological changes on day 5 and it becomes minimal in 42 days PI e. All model showed seronegative against SARS-CoV-2 f. They developed neutralizing antibody as early as 7 days after infection g. After reinfection, the viral was not found in any cats	[13]
Animal model	Cat (4)	AI, NI	1 – 10	a. Virus was detected from day 1 to 6 PI along to the clinical signs b. The virus can be transmitted through contact through cohoused c. There is a potential of human-cat-human transmission	[14]
Animal model	Tree shrew (38)	AI	2, 4, 6, 7, 8, 10, 12, 14, 16	a. There is an increase in body temperature in tree shrews until the day 8 PI b. An increase in body temperature occurs more severely in old tree shrews c. RNA virus was detected on the day 6 PI in several specimens, including nasal, throat, an anal swab, and blood d. Viral shedding gradually decreased on the day 12 PI in young tree shrews, however not in the old groups e. Moderate pneumonia occurs in all groups, and mild histopathological change occurs in the brain, liver, pancreas, and heart f. SARS-CoV-2 caused asymptomatic infection in young, and affect men severely than a woman	[15]
Animal model	Swine (18)	AI, NI	1, 4, 8, 21	a. SARS-CoV-2 was able to replicate in swine cell lines and causes cytopathic effects b. This virus did not cause any clinical signs in swine, viral shedding, and antibody responses c. Pig is unlikely to act as the reservoir of SARS-CoV-2	[16]
Surveillance	Pig (187), cow (107), sheep (133), horse (18), chicken (153), duck (153), goose (25), mice (81), rat (67), guinea pig (30), rabbit (34), monkey (39), dog (487), cat (87), wild animal (313), ferret (1)	-	-	All serum samples from collected animals were negative regarding its antibody against SARS-CoV-2	[17]
Surveillance	Cat (9), dog (12)	NI	-	SARS-CoV-2-specific antibody was not detected in those animals even have repeated contact with the infected human	[18]
Surveillance	Cat (920)	-	-	a. There is 0.69% (6/920) serum samples were positively detected contain antibody against SARS-CoV-2 b. High incidence of human infected with SARS-CoV-2 in German has low transmission to cats population c. It indicated that there is a human-cat transmission regarding SARS-CoV-2, however, it does not prove that there is a circulation of this virus among the cat population	[19]
Surveillance	Cat (50)	-	-	a. There are 6 (12%) cases of human-cat transmission regarding SARS-CoV-2 b. Only one sample has an identic virus genome sequence with the owner c. There is a potential of transmission but very low	[20]
Case report	Pomeranian dog (1), German Shepherd (1)	NI	Varies from 1-13	a. RNA virus was detected from a nasal and oral swab in Pomeranian dog (days 2, 5, and 9), negative on day 12, and reported dead on day 15 b. RNA virus was detected from a nasal and oral swab in German Shepherd (days 1 and 2), negative on day 3	[21]
Case report	Cat (2)	NI	-	a. Cat A showed clinical signs including sneezing, ocular discharge, and mild lethargy that confirmed positive SARS-CoV-2 infection. Cat A is fully recovering in 8 days AD b. Cat B showed similar clinical signs to cat A, and fully recovered in 2 days AD c. It is suspected that cat can produce specific antibody against SARS-CoV-2 infection	[22]

(-)=unclearly understood, AI=Artificial infection, NI=Natural infection/direct contact with an infected population, PI=Post-infection, AD=After diagnosed positive. SARS-CoV-2=Severe acute respiratory syndrome-coronavirus 2

### Trend of diagnostic tools used in previous studies

**Figure-2:**
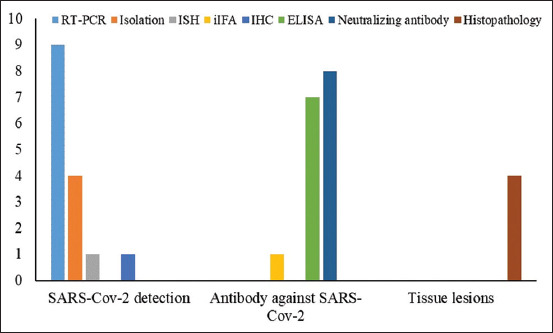
Trend of detection tools used in the studies that merit to the criteria.

Among the 12 articles that met our criteria, there were several diagnostic methods used in the detection of SARS-CoV-2. However, the use of those methods was determined on the basis of the target of detection. There are three targets of detection: Viral representation, antibodies, and tissue lesions. The most common methods used for SARS-CoV-2 RNA detection were reverse transcriptase-polymerase chain reaction (RT-PCR), viral isolation, *in situ* hybridization (ISH), indirect immunofluorescence assay (iIFA), and immunohistochemistry (IHC). The microneutralization assay and enzyme-linked immunosorbent assay (ELISA) were commonly used in the detection of antibody synthesis by the host body. Histopathology was the only procedure in the detection of tissue lesions. The details of diagnostic methods used in SARS-CoV-2 studies are embedded in Figure-2.

### Histopathology and clinical signs

**Figure-3:**
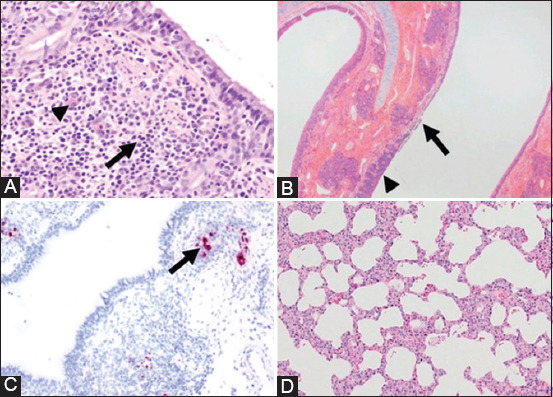
Histopathological feature from animals infected by severe acute respiratory syndrome-coronavirus 2 (SARS-CoV-2). Moderate infiltration of lymphocyte (arrow) and plasma cell (arrowhead) within the submucosa of bronchi from a cat on 7 days post infection (DPI) (A); mucosa ulceration and epithelial hyperplasia of nasal turbinate from a cat on 5 DPI (B); expression of SARS-CoV-2 RNA in cat’s submucosal bronchi (arrow) on 4 DPI (C); normal histological feature of swine’s lung on 4 DPI (D). H&E, 200× (A) [11], 40× (B) [13], 10× (D) [16]; immunohistochemistry, 100× (C) [11].

From those studies, there were several histopathological changes observed from SARS-CoV-2 infection within the animal, such as multifocal lymphocytic and neutrophilic tracheobronchoadenitis [11], moderate ulcerative and suppurative lymphoplasmacytic rhinitis [13], mild-to-moderate changes in the liver and kidney, and, surprisingly, mild changes in the brain [15]. Histopathological changes occurred predominantly within the respiratory system, whereas the other organs were normal. IHC showed that the RNA of SARS-CoV-2 was observed on days 4-7 after infection, whereas this was not observed on day 21 [11]. However, the previous studies reported that histopathological changes occurred predominantly in cats and did not cause any clinical signs (asymptomatic). By contrast, there were no histopathological changes observed in infected swine [16]. Unfortunately, the histopathology observed in these studies was only seen in those using artificial infection in animals and not in the surveillance and case reports that were caused by natural infection. Several histopathological changes found in this review are embedded in Figure-3[11,13,16].

### Viral shedding

Viral shedding was found in mainly the specimen of artificial infection studies rather than in specimens with natural infection. We observed that 57.14% (4/7) of studies detected the RNA of SARS-CoV-2 from respiratory organs [11,12,14,15,21]. Only one study reported viral shedding from natural infection [21]. Moreover, 33.33% (1/3) of studies demonstrated the presence of specific antibodies against SARS-CoV-2, but with minimal concentration [19], whereas the rest of the studies showed absence [17,18]. The phylogenetic analysis of a previous study reported that the potential of human-cat transmission of SARS-CoV-2 is minimal, with a low similarity (16.66%; 1/6) of the viral genome sequence between cohoused humans and cats [20]. No other research report in PubMed® published in 2020 has demonstrated the similarity of the SARS-CoV-2 sequence in humans and animals.

### Potential of transmission

The number of articles that analyzed the similarity of the viral sequence among humans and animals is limited. Nevertheless, several articles within the scope of our observation contained information regarding the potency of SARS-CoV-2 transmission. In this study, the potential of transmission was reported using the conjecture of shortest period of transmission, where the virus was first detected, and the history of comingling with animals positive for the SARS-CoV-2 infection. A report regarding the potential of transmission is embedded in Table-3 [11-14,20-22].

**Table-3:** Potential of SARS-CoV-2 transmission reported by the previous studies.

Potential of transmission	Shortest period of transmission	Site where the virus was first detected		Reference

		Respiratory tract	Non-respiratory tract	
Cat-cat	2 days	N, OP,	R	[11]
Cat-cat	3 days	N, SP, Tc	Ts	[12]
Cat-cat	1 day	N, Tc	E	[13]
Cat-cat	2 days	N	-	[14]
Human-cat	1 day	N, Or	R	[20]
Human-dog	2 days	N	-	[21]
Human-cat	8 days	N	-	[22]

N=Nasal, OP=Oropharyngeal, R=Rectal, SP=Soft palate, Tc=Trachea, Ts=Tonsil, E=Esophagus, Or=Oral. SARS-CoV-2=Severe acute respiratory syndrome-coronavirus 2

### Correlation between potential factors

Inhabitation was not correlated with viral shedding in pet cats (p≥0.05). Comingling did not affect the representation of SARS-CoV-2 viral shedding among pet cats, with an RR of 0.88 (95% CI: 0.82-0.93). However, the inhabitation potentially affected the representation of viral shedding in pet dogs (p≤0.05) with an RR of 0.93 (95% CI: 0.85-1.02). Few instances of viral shedding were seen in pet cats and dogs that live with their owner or with animals infected with SARS-CoV-2 (Table-4). Viral shedding of SARS-CoV-2 was correlated to the clinical signs occurring in pet cats and dogs (p≤0.05) (Table-5). Furthermore, SARS-CoV-2 viral shedding in cats is usually asymptomatic. The RR of viral shedding in asymptomatic cats was 0.86 (95% CI: 0.71-1.05), whereas that in dogs was 0.33 (95% CI: 0.06-1.65). This proves the high potency of SARS-CoV-2 to cause asymptomatic clinical signs in cats. Another study found a correlation between viral shedding and the synthesis of neutralizing antibodies against SARS-CoV-2 in both cats and dogs (Table-6). Pet cats and dogs generated neutralizing antibodies against SARS-CoV-2 with an RR of 0.43 (95% CI: 0.25-0.76) and 0.50 (95% CI: 0.12-1.99), respectively. Thus, SARS-CoV-2 infection may have a better prognosis in pets because of their ability to synthesize antibodies in a short period of time.

**Table-4:** Correlation between inhabitation (comingled and separated) to the SARS-CoV-2 viral shedding among the companion animals.

Animal species	Inhabitation	Viral shedding		n	χ^2^	p-value

		Positive	Negative			
Cat	Comingled	16	119	156	2.77	0.96
	Separated	0	21			
Dog	Comingled	2	29	130	6.48	0.01*
	Separated	0	99			

* There is a correlation due to p*≤*0.05. SARS-CoV-2=Severe acute respiratory syndrome-coronavirus 2

**Table-5:** Correlation between SARS-CoV-2 viral shedding to the clinical signs among the companion animals.

Animal species	Viral shedding	Clinical signs		n	χ^2^	p-value

		Symptomatic	Asymptomatic			
Cat	Positive	2	13	68	7.28	0.00*
	Negative	0	53			
Dog	Positive	2	1	14	14.00	0.00*
	Negative	0	11			

* There is a correlation due to p*≤*0.05. SARS-CoV-2=Severe acute respiratory syndrome-coronavirus 2

**Table-6:** Correlation between SARS-CoV-2 viral shedding to the neutralizing antibody among the companion animals.

Animal species	Viral shedding	Neutralizing antibody		n	χ^2^	p-value

		Positive	Negative			
Cat	Positive	9	7	135	71.71	0.00*
	Negative	0	119			
Dog	Positive	1	1	31	14.98	0.00*
	Negative	0	29			

* There is a correlation due to p*≤*0.05. SARS-CoV-2=Severe acute respiratory syndrome-coronavirus 2

## Discussion

SARS-CoV-2 is the seventh coronavirus that can infect humans and cause respiratory failure that leads to death. On its surface, they develop various spikes to support the attachment on the host cells. One of the potential virulence factors of SARS-CoV-2 is a receptor-binding domain (RBD) [23]. This RBD is suspected to undergo a mutation that increases its affinity to the angiotensin-converting enzyme-2 (ACE-2) [24]. The RBD has six different amino acids: F486, L455, N501, Q493, S494, and Y505 [25]. These RBDs have a high affinity to bind to ACE-2 in humans and animals with high receptor homology. Nowadays, more than 120 million people worldwide have been infected with SARS-CoV-2. Bats are suspected to act as the primary reservoir of SARS-CoV-2. A previous study reported the rich diversity of SARS-like virus in the area with a high population of bats [26]. However, the transmission of SARS-CoV-2 from companion animals to humans is still unknown and has caused social panic during the pandemic in 2020.

In PubMed, there are a few (n=132) published articles describing SARS-CoV-2 infection in animals. Unfortunately, only 9.09% (12/132) of these articles were reports of either artificial or natural infection among the animals (Table-1). Thus, there are a limited number of real-world reports on the occurrence of SARS-CoV-2 infection in animals. The review articles found in PubMed were limited to explaining general knowledge regarding coronaviruses; only a few focused on real cases of SARS-CoV-2 infection in animals. The limited references and limited supporting evidence with the high speed of unfiltered information on the internet trigger the development of public opinion regarding the potential of SARS-CoV-2 transmission from animals (especially companion animals) to humans. For example, the first report of SARS-CoV-2 infection in the tiger and lion of Bronx Zoo caused panic abandonment of pets such as cats and dogs. During the involvement of public health experts during the investigation, the SARS-CoV-2 genome sequence of the infected tiger was identical to that of the tiger’s zookeepers, which was confirmed previously before the animal was infected [6]. This proves the presence of human-animal transmission (reverse zoonosis) [20]. Human-to-animal viral transmission in the previous studies was detected using several procedures.

Until now, testing for SARS-CoV-2 involves the use of RT-PCR as a routine procedure [27], which has higher sensitivity than the other methods. Furthermore, several studies have reported other diagnostic tools for SARS-CoV-2 detection, including serology [28], ELISA [29], and IHC [30]. However, each method of detection has its limitations. For example, the PCR be used directly by end-users because this method needs qualified technicians and complex laboratory equipment. These complex requirements of RT-PCR can lead to false results if errors happen during sample collection, storage, transfer, and sample processing [31]. Viral isolation must be conducted through a sterile procedure [32]. ISH and IHC must have optimization protocols with a perfect blocking procedure and organ suitability, and they can sometimes be combined [33]. Regarding the serological tests in the previous studies, both ELISA and iIFA, despite being optimized for cross-reactivity, have low sensitivity and inconsistency in the detection of SARS-CoV-2 antibodies because of the limited concentration of the synthesized antibody [34]. Similar to PCR, histopathology needs an expert to conduct the tissue assessment [35]. Based on our findings, the limitations of each test should be considered when utilizing a single method in the evaluation of SARS-CoV-2, whether experimentally or clinically. When screening of SARS-CoV-2 in suspected patients/hosts, a combination of tests can be conducted to cover the weaknesses of each test. Nevertheless, it is worth noting that the limit of detection is also influenced by viral load and the presentation of histopathological changes.

The histopathological changes caused by SARS-CoV-2 infection in domesticated animals commonly occur in the respiratory system. These changes ­predominantly include lymphocyte and neutrophil infiltration, such as lymphocytic-neutrophilic tracheobronchoadenitis. In a cat, neutrophil and lymphocyte infiltration occurred in the first stage of infection (days 1-7) [11], but in this case, the main cluster of lymphocytes that infiltrated the tissue was not specified. Based on the other report in the human case, all types of lymphocytes decreased during SARS-CoV-2 infection, and the increase of CD8+ and CD4+ was correlated with good prognosis and clinical efficacy of treatments [36]. A previous study described that neutrophil and lymphocyte infiltration synergistically occurred with the presentation of SARS-CoV-2 RNA within the cat’s infected tissue [11]. Another study reported that the decrease of neutrophil-to-lymphocyte ratio (NLR) increases the rate of healing and chances of cure [37]. By contrast, increased NLR is associated with poor clinical outcomes and death [37,38]. The increase of NLR causes microvascular obstruction [39]. In advanced diseases, the increase of NLR influences the release of inflammatory cytokines, resulting in multiorgan failure [40]. However, massive histopathological changes are seen in humans rather than in cats. It has been reported that the lungs of infected patients undergo diffuse alveolar damage, vascular thrombosis, and endothelialitis, which eventually cause acute respiratory distress syndrome and death [41]. Another report showed lymphocytic myocarditis, acute tubular injury, microthrombi, ischemic necrosis, hemophagocytosis, and deep vein thrombosis in humans infected with SARS-CoV-2 [42]. By contrast, those histopathological changes were not observed in experimental cats [11,13], tree shrews [15], and swine [16]. The minimal lesions and good prognosis in animals infected with SARS-CoV-2 indicate that they can generate immunity against infection and viral load.

Viral load pertains to the amount of virus within an organism, typically in the bloodstream. This impacts the occurrence of clinical signs [43]. However, in SARS-CoV-2 infection, viral load within the upper respiratory system is associated with the presence of clinical signs [44]. Both humans and animals show similar disease progression and pathogenesis. However, the virus can be eliminated faster in cats than in humans. A previous study described that viral load in cats can be eliminated during days 14-21 after it is first detected in the upper respiratory system [11]. By contrast, a longer period of elimination was observed in humans [45]. In general, the peak viral load of SARS-CoV-2 occurs within a week of symptoms appearing. At the same time, the virus can be transmitted through respiratory discharge and sputum [11,14]. In this review, we found that humans and cats have similar pathogenesis regarding viral replication within the upper respiratory system during the 1^st^ week. However, these trends differed in the 2^nd^ week, with the viral load increasing in humans but decreasing in animals. This was followed by a good prognosis in cats but not in humans. Several studies reported that SARS-CoV-2 cannot replicate within swine [16], cattle, goat, sheep, and poultry [17]. The ability of SARS-CoV-2 to replicating inside humans and cats occurs because of their similarities in ACE-2 receptors [46].

The ACE-2 receptor, which is expressed within the lung, plays a significant role in converting angiotensin II to become angiotensin I, protecting the lung from acute injury [47], and in the hydrolysis of proline [48]. Loss of ACE-2 expression within the lung tissue can cause vascular permeability, lung edema, and accumulation of neutrophils during acute injury, as well as interfere with normal lung function [49]. Recently, ACE-2 has been suspected to act as the specific receptor for SARS-CoV-2. This is because the spike protein of SARS-COV-2 is highly similar to that of the previous coronavirus (SARS-CoV) [25]. ACE-2 not only acts as the receptor but also potentially increases the risk of human-human transmission. Because of the increased risk of zoonosis and public health concerns, a molecular analysis of the ACE-2 in animals has been conducted. An animal study found that ACE-2 binds to the S protein of several coronaviruses, whereas this affinity is different in humans [50]. When the human ACE-2 interacts with the S protein of SARS-CoV, it can cause an infection. By contrast, the infection was nearly absent in animals, especially civets and rats. The high infectivity in humans after ACE-2 binds to the S protein of the coronavirus is caused by the alteration of histidine 353 to human lysine. This alteration interferes with the S protein-mediated infection and increases the affinity of the RBD [51]. These findings are supported by a previous study which found that both humans and rhesus monkeys generate the most efficient receptor for SARS-CoV-2, whereas other animals (i.e., canine, rabbit, feline, and pangolin) showed >50% potency and the rest (i.e., rat and mouse) indicated very low potency [52]. Furthermore, it is implied that SARS-CoV-2 can potentially transmit from human to human and human to animal either through artificial or natural exposure.

Because of these findings, companion animals such as cats and dogs have become important to study because of their high interaction with humans. A previous study found that cats and dogs expressed ACE-2 within their kidney, myocardial [53], and lung tissue [54]. The ACE-2 of cats showed high similarity to that of humans in terms of their amino acids [55]. Cats and dogs support pseudotypes of SARS-CoV-2, thus raising the possibility of viral transmission. We found cat-cat, human-cat, and human-dog transmission in 4/7, 2/7, and 1/7 studies, respectively (Table-3). However, no correlation was found between inhabitation and viral shedding in cats. Nevertheless, it should be noted that the period of exposure can influence this result [56]. Conversely, pet dogs showed a higher probability of testing positive after comingling with the owners. This indicates that pet cats can eliminate the virus in a shorter period of time by synthesizing neutralizing antibodies against SARS-CoV-2 when compared with pet dogs. Surprisingly, both pet cats and dogs were asymptomatic despite the detection of viral shedding. This could be attributed to immune formation, such as the production of neutralizing antibodies synthesized by cats and dogs. Pet cats and dogs can generate neutralizing antibodies against SARS-CoV-2 on day 5, which is faster when compared with humans [11] that synthesize antibodies on day 14, peaking on day 28 [57]. The correlation between neutralizing antibodies and viral shedding indicates that a faster antibody synthesis can prevent worse clinical manifestations. In current medical therapy, neutralizing antibodies are used as treatment against SARS-CoV-2 infection, with a good prognosis [58].

This analysis proved that pet cats and dogs can be infected with SARS-CoV-2. However, no study published in PubMed during 2020 declared and observed the potential of transmission of SARS-CoV-2 from pet cats and dogs to humans (zoonosis). The mode of transmission of those animals to humans is still complicated and needs further exploration. Nevertheless, we found that transmission occurred from humans to cats and dogs (zooanthroponosis). Thus, it is still possible that those animals act as silent intermediate hosts of SARS-CoV-2 because of the representation of ACE-2 within their tissue. However, the panic abandonment of such pets should be stopped because there is currently no scientific proof supporting viral transmission from companion animals to humans. The asymptomatic clinical signs present in pet cats and dogs cannot be used as the main indicator for animal to human transmission. Animal discrimination during this 2^nd^ year of the pandemic should be banned to uphold animal welfare. Veterinary and human health experts must have a united approach in the management of the SARS-CoV-2 global pandemic. Routine surveillance should be conducted to evaluate the susceptibility of this virus in pets as well as in wild animals. Good management practice in maintaining such pets is the most suitable way to prevent companion animals from SARS-CoV-2 infection [59].

## Conclusion

SARS-CoV-2 transmission in domesticated animals is frequently found, especially in pet cats and dogs compared with other animals. SARS-CoV-2 can be transmitted between the same species (cat-cat). Reverse zoonosis (zooanthroponosis) was found from humans to cats/dogs that comingled with their infected owners. These animals were generally asymptomatic because of the fast formation of neutralizing antibodies, but viral shedding was still seen. Moreover, comingling with infected humans was correlated with both viral transmission in dogs and the presence of clinical signs. Further research regarding SARS-CoV-2 transmission in animals with larger sample size and better quality is needed to validate our findings regarding its disease pathogenesis and transmission. These will also be useful to better understand the dynamics of zoonosis and zooanthroponosis dynamics from a public health perspective.

## Authors’ Contributions

YAP and YPK: Concept of study and performed analysis. YAP, YPK, CSR, DW, MS, and NH: Drafted and revised the manuscript. All authors read and approved the final manuscript.
